# Effects of clinical and environmental factors on bronchoalveolar antibody responses to *Pneumocystis jirovecii*: A prospective cohort study of HIV+ patients

**DOI:** 10.1371/journal.pone.0180212

**Published:** 2017-07-10

**Authors:** Robert J. Blount, Kieran R. Daly, Serena Fong, Emily Chang, Katherine Grieco, Meredith Greene, Stephen Stone, John Balmes, Robert F. Miller, Peter D. Walzer, Laurence Huang

**Affiliations:** 1 Division of Pulmonary and Critical Care Medicine, University of California, San Francisco, California, United States of America; 2 Division of Pediatric Pulmonology, University of California, San Francisco, California, United States of America; 3 Division of Infectious Diseases, University of Cincinnati, Cincinnati, Ohio, United States of America; 4 Veterans Administration Medical Center, Cincinnati, Ohio, United States of America; 5 HIV/AIDS Division, San Francisco General Hospital, University of California, San Francisco, California, United States of America; 6 Environmental Health Sciences, University of California, Berkeley, California, United States of America; 7 Research Department of Infection and Population Health, Institute of Epidemiology and Healthcare, University College London, London, United Kingdom; 8 Department of Infectious and Tropical Diseases, London School of Hygiene and Tropical Medicine, London, United Kingdom; Medical University of South Carolina, UNITED STATES

## Abstract

**Background:**

Humoral immunity plays an important role against *Pneumocystis jirovecii* infection, yet clinical and environmental factors that impact bronchoalveolar antibody responses to *P*. *jirovecii* remain uncertain.

**Methods:**

From October 2008—December 2011 we enrolled consecutive HIV-infected adults admitted to San Francisco General Hospital (SFGH) who underwent bronchoscopy for suspected *Pneumocystis* pneumonia (PCP). We used local air quality monitoring data to assign ozone, nitrogen dioxide, and fine particulate matter exposures within 14 days prior to hospital admission. We quantified serum and bronchoalveolar lavage fluid (BALF) antibody responses to *P*. *jirovecii* major surface glycoprotein (Msg) recombinant constructs using ELISA. We then fit linear regression models to determine whether PCP and ambient air pollutants were associated with bronchoalveolar antibody responses to Msg.

**Results:**

Of 81 HIV-infected patients enrolled, 47 (58%) were diagnosed with current PCP and 9 (11%) had a prior history of PCP. The median CD4+ count was 51 cells/μl (IQR 15–129) and 44% were current smokers. Serum antibody responses to Msg were statistically significantly predictive of BALF antibody responses, with the exception of IgG responses to MsgC8 and MsgC9. Prior PCP was associated with increased BALF IgA responses to Msg and current PCP was associated with decreased IgA responses. For instance, among patients without current PCP, those with prior PCP had a median 73.2 U (IQR 19.2–169) IgA response to MsgC1 compared to a 5.00 U (3.52–12.6) response among those without prior PCP. Additionally, current PCP predicted a 22.5 U (95%CI -39.2, -5.82) lower IgA response to MsgC1. Ambient ozone within the two weeks prior to hospital admission was associated with decreased BALF IgA responses to Msg while nitrogen dioxide was associated with increased IgA responses.

**Conclusions:**

PCP and ambient air pollutants were associated with BALF IgA responses to *P*. *jirovecii* in HIV-infected patients evaluated for suspected PCP.

## Introduction

*Pneumocystis* pneumonia (PCP), a disease first clinically described in premature and malnourished children in the 1940s [1] and later found to be an important opportunistic infection among immunosuppressed patients such as those with HIV infection, continues to impart significant morbidity and mortality worldwide [2].

*Pneumocystis* infection is cleared mainly through cell-mediated immunity although humoral immune processes are also vital [3–11]. For instance, in animal studies B cell deficient transgenic mice died from *Pneumocystis* infection more rapidly than B cell competent controls [12], and in human case reports, humoral immune deficiencies such as X-linked agammaglobulinemia were the sole PCP risk factors identified [13–15]. Given the clinical importance of humoral responses to *Pneumocystis* and the difficulties propagating *Pneumocystis* in culture [16], we have developed recombinant fragments of the *Pneumocystis jirovecii* major surface glycoprotein (Msg) and enzyme-linked immunosorbent assays (ELISA) to detect antibody responses to Msg [17–20]. Emerging research on *P*. *jirovecii* serological markers has contributed to PCP diagnostics [21, 22] and to a better understanding of the clinical [23–25] and environmental influences [26–29] on the organism and host antibody responses.

*Pneumocystis* infection and pathology are usually localized to the lungs yet little is known about the local antibody responses to *P*. *jirovecii* and what clinical and environmental factors drive these responses [3, 30, 31]. Tobacco smoking and ambient air pollution may influence PCP presentation and serum antibody responses to *P*. *jirovecii* [27, 28], yet it remains unknown how inhaled pollutants impact bronchoalveolar antibody responses to Msg. In response to these knowledge gaps we formulated the following research questions: in patients with HIV, 1) are serum antibody responses to *Pneumocystis* Msg reflective of bronchoalveolar antibody responses to Msg, 2) how do prior PCP, current PCP, and degree of immunosuppression impact bronchoalveolar antibody responses, 3) what are the influences of air pollutants on bronchoalveolar antibody responses to *Pneumocystis* Msg, and 4) are bronchoalveolar antibody responses to *Pneumocystis* predictive of clinically important outcomes? To address these questions we enrolled hospitalized HIV patients undergoing bronchoscopy for suspected PCP into a prospective cohort study, following them throughout their hospital admission and determining serum and bronchoalveolar antibody responses to *P*. *jirovecii* Msg.

## Methods

### Study population

From October 2008—December 2011, we enrolled consecutive HIV-infected adults hospitalized at San Francisco General Hospital (SFGH) who underwent bronchoscopy with bronchoalveolar lavage for suspected PCP. These patients were concurrently enrolled into the International HIV-associated Opportunistic Pneumonias (IHOP) Study, a previously described longitudinal cohort study of HIV-infected adults with clinical and radiographic evidence of pulmonary infection [2, 32]. We included those who had PCP confirmed or ruled out by modified Giemsa staining of bronchoalveolar lavage fluid (BALF) and who lived within the San Francisco city limits. We excluded those lacking capacity to participate in the consent process (from delirium, dementia, or psychiatric conditions) and those from whom we did not have sufficient BALF for antibody testing. We followed participants throughout their stay in the hospital and for two months following hospital discharge. We obtained clinical and demographic data through direct patient interview using standardized questionnaires and through chart abstraction of hospital and HIV outpatient clinic paper and electronic medical records using a standardized chart abstraction form. We then entered clinical and demographic data into a secure Research Electronic Data Capture (REDCap, Harvard Catalyst, USA) database using multiple validation tools to ensure accuracy.

### Bronchoalveolar lavage

Bronchoalveolar lavage was performed for clinical indications following British Thoracic Society guidelines for fiberoptic bronchoscopy in adults [33]. The bronchoscope was directed into the most affected sub-segment(s) identified by chest imaging. Once the tip of the bronchoscope was gently wedged into a distal airway, 20 ml of 0.9% sterile saline was instilled and recovered by gentle aspiration. Serial 20 ml aliquots were instilled and recovered for a goal of 50% recovery of approximately 40 ml BALF. This freshly obtained BALF was placed on ice at the bedside and immediately transported to the lab. Specimens were centrifuged and 1 ml aliquots of supernatant were cryopreserved at -80°C awaiting Msg analysis.

### Recombinant antigen preparation

As previously described [18, 25], using PCR with AmpliTaq enzyme (Applied Biosystems, Carlsbad, CA, USA) we amplified 3 overlapping segments, Msg_15-1119_, Msg_729-2282_, and Msg_2015-3332_, of the entire Msg gene derived from *P*. *jirovecii* infected human lung. We inserted these segments into pET30 *E*. *coli* expression systems (Novagen, Madison, WI, USA) to generate corresponding Msg proteins: MsgA (amino-terminus), MsgB (middle portion), and MsgC1 (carboxyl terminus). As the Msg carboxyl-terminus has been found to be the most antigenically conserved region of the Msg protein [17], we created three additional recombinant proteins from this region: MsgC3, MsgC8, and MsgC9 [20]. Proteins were purified by affinity chromatography.

### Measurement of antibody responses to Msg

We used previously developed ELISA protocols to quantify IgM and IgG serologic responses to the aforementioned recombinant Msg constructs [24]. Similarly, to determine IgA, IgM, and IgG responses to Msg in BALF, we tested BALF and standard reference specimens against each Msg construct, using phosphate-buffered saline (PBS) without Msg as the negative control, correcting the reactivity of each BALF specimen to Msg by subtracting the mean optical density with PBS alone from the mean optical density with Msg [34]. As previously described, we generated a standard curve for each Msg construct on each day that the assay was performed, and used this curve to calculate units of reactivity [25]. We diluted BALF samples at 1:5 to 1:10 to fit the linear portion of the standard curves. Taking into account the dilution, we then calculated units of reactivity. Of note, at the time of this analysis, IgA protocols had only been developed for BALF to MsgA, MsgC1, and MsgC8, but not against the other Msg constructs, and not for serum. For this study, BALF was diluted 1:5, and serum 1:100 prior to analysis. Antibody responses reported in the results and tables were not corrected for the dilution factor.

### Ambient air pollutant measurements

We determined daily mean concentrations of particulate matter < 2.5 μm in diameter (PM_2.5_) (μg/m^3^), daily maximum 1-hour nitrogen dioxide (NO_2_) concentrations in parts per billion (ppb), and daily maximum 8-hour ozone (O_3_) concentrations (ppb) at the single, centrally located monitoring station in San Francisco on 10 Arkansas Street operated by the Bay Area Air Quality Management District in compliance with U.S. Environmental Protection Agency regulations. Ambient air pollutant exposures were assigned for each of the 14 days prior to each participant’s hospital admission for suspected PCP. We chose this time frame *a priori* given that this is the typical subacute time course from subclinical to fully symptomatic PCP in which lung antibody responses to *P*. *jirovecii* infection are mounting [2, 7, 35, 36].

### Ethical considerations

The study was approved by the institutional review boards at the University of California, San Francisco and the University of Cincinnati. Written informed consent was obtained from each participant.

### Statistical analyses

For descriptive statistics of baseline characteristics Student’s t-test was used for continuous variables and Chi-squared and Fisher’s exact testing for categorical variables. To test the correlation between serum and BALF antibody responses, the Pearson correlation coefficient *r* was used and those with undetectable antibody responses were excluded from the analysis. Potential confounders were selected *a priori* based on their biologically plausible association with both predictors and outcomes and included age, sex, race, ethnicity, CD4 cell count, HIV RNA viral load, receipt of antiretroviral therapy or *Pneumocystis* prophylaxis at the time of admission, a prior history of PCP, current tobacco smoking (within the two months prior to admission), and homelessness. Potential confounders that were associated with outcome at a level of statistical significance of *P* < 0.2 in bivariate analyses were included in final multivariable regression analyses. We tested the association between serum and BALF antibody responses to each Msg construct using multivariable linear regression analyses excluding those with undetectable antibody responses. We tested the association of BALF IgA responses to Msg with a current diagnosis of PCP using multivariable Tobit regression analyses specified for left censored data, stratifying by prior PCP history. We tested for effect modification by defining interaction terms between prior PCP and IgA responses to each of the following Msg: MsgA, MsgC1, and MsgC8, fitting separate multivariable Tobit regression models with each interaction term and reporting effect modification for a *P* interaction ≤ 0.05. Similarly, separate multivariable Tobit regression analyses were used to test the associations between each pollutant (scaled to 10 μg/m^3^ PM_2.5_, 10 ppb of NO_2_, and 10 ppb of O_3_) on each of the 14 days prior to admission and BALF IgA responses to Msg. Results were reported as β coefficients representing the change in BALF IgA (U) predicted by a 10 unit increase in pollutant, along with 95% CIs and *P* values. To test the influence of ambient air pollutants and antibody responses on clinical outcomes multivariable linear regression models were fitted with the predictors (ambient air pollutant levels and BALF IgA responses to Msg constructs) and the outcome (length of hospital admission). Multivariable Cox models were fitted to test associations of the same predictors with 2-month mortality.

## Results

### Baseline characteristics

**Fig 1:**
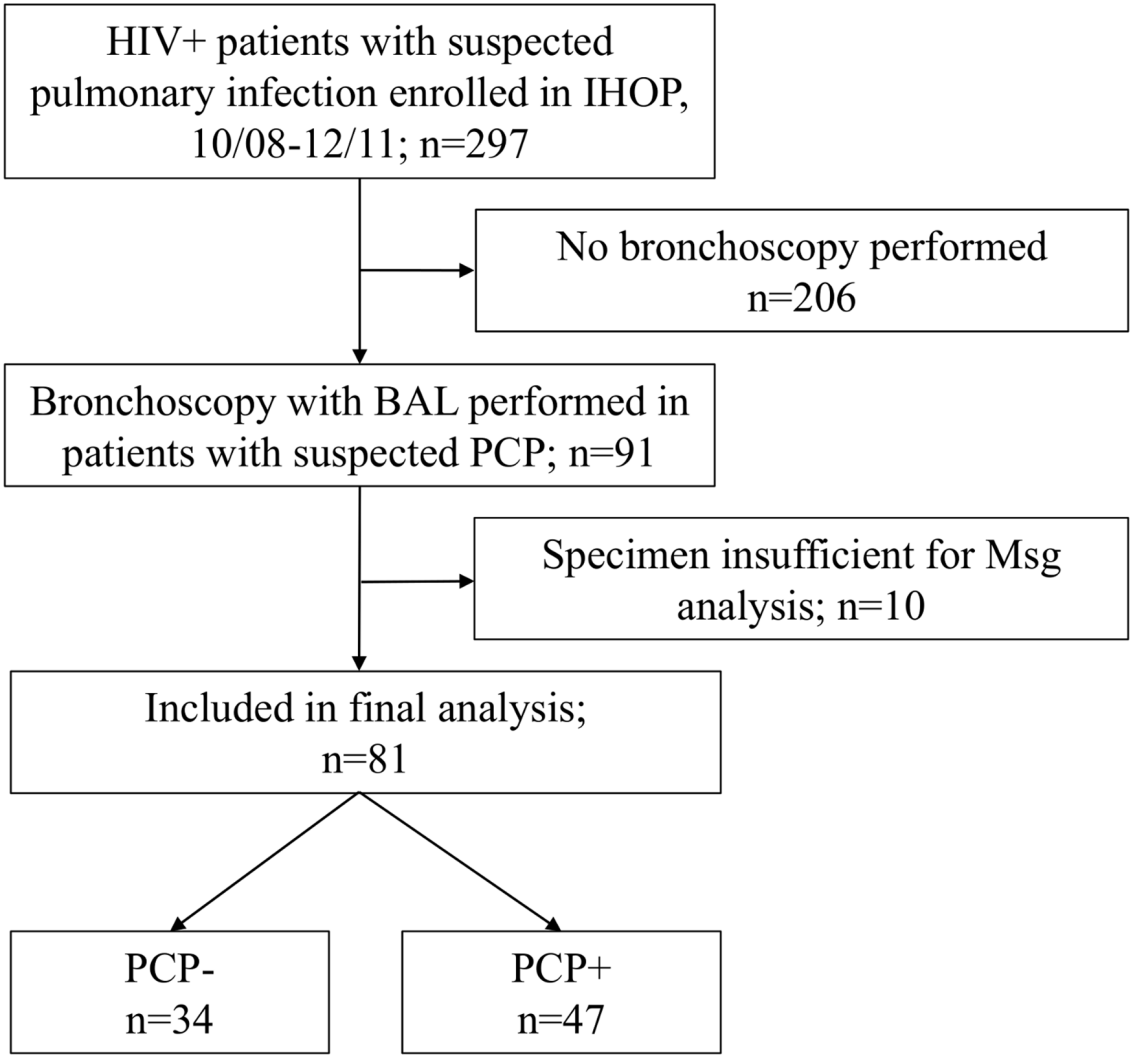
Enrollment flow chart. Abbreviations: IHOP, International HIV-associated Opportunistic Pneumonias Study; BAL, bronchoalveolar lavage; PCP, *Pneumocystis* pneumonia; Msg, recombinant *Pneumocystis jirovecii* major surface glycoprotein.

We enrolled 297 HIV-infected patients with suspected pulmonary infection into the IHOP prospective cohort study between October 2008 and December 2011 (Fig 1). From this cohort, bronchoscopy with bronchoalveolar lavage was performed in 91 patients with suspected PCP. Ten of these patients were excluded because the quantity of BALF, after use for clinical diagnostics, was insufficient for Msg analysis. The remaining 81 patients, all San Francisco residents, were included in the final analysis: 47 with laboratory-confirmed PCP and 34 without PCP. Additional pulmonary infections diagnosed included bacterial or viral community acquired pneumonia (n = 36, 44%); health care associated, hospital acquired, or ventilator associated pneumonia (n = 4, 4.9%); pulmonary *Mycobacterium avium* complex (MAC) infection (n = 3, 3.7%); non-PCP fungal pneumonia (n = 2, 2.5%), and active pulmonary tuberculosis (n = 1, 1.2%). Multiple pulmonary infections were present in 15 patients with PCP (32%) and two patients without PCP (5.9%). Noninfectious causes of respiratory symptoms included Kaposi’s sarcoma (n = 5, 6.2%); asthma and/or chronic obstructive pulmonary disease (COPD) (n = 3, 3.7%); pulmonary emboli (n = 1, 1.2%); and pulmonary edema (n = 1, 1.2%).

Participants were predominantly middle-aged (median 46 years, IQR 39–50), male (89%), and white (48%) (Table 1). Nearly half of patients were current tobacco smokers, and 19% were homeless. Among patients with PCP, CD4 counts were lower (median 45 vs. 72 cells/μl), and viral loads were higher (5.25 vs. 4.35 log_10_ copies/ml) when compared to patients without PCP (these difference did not reach statistical significance *P* = 0.07 and 0.13, respectively). Patients without PCP compared to patients with PCP were statistically significantly more likely to be taking antiretroviral therapy (74% vs 11%, *P* < 0.001) and PCP prophylaxis (31% vs. 6.4%, *P* = 0.003).

**Table 1:** Baseline characteristics by PCP diagnosis, n = 81.

Characteristic	Total	PCP-n (%)*	PCP+n (%)	*P* value
N	81	34	47	
Age, median (IQR), years	46 (39–50)	46 (42–54)	45 (38–50)	0.16
Male	72 (89)	30 (88)	42 (89)	0.87
Race/Ethnicity				0.77
White	39 (48)	14 (41)	25 (53)	
Black	21 (26)	11 (32)	10 (21)	
Hispanic	13 (16)	5 (15)	8 (17)	
Asian	6 (7.4)	3 (8.8)	3 (6.4)	
Native American	2 (2.5)	1 (2.9)	1 (2.1)	
CD4 count, median (IQR), cells/μl	51 (15–129)	72 (14–220)	45 (16–100)	0.07
Viral load (log_10_ copies/ml)	5.10 (4.31–5.50)	4.35 (1.87–5.28)	5.25 (4.86–5.70)	0.13
Antiretroviral therapy^†^	28/76 (37)	**23/31 (74)**	**5/45 (11)**	**<0.001**
PCP prophylaxis^†^	13/79 (16)	**10/32 (31)**	**3/47 (6.4)**	**0.003**
Prior PCP	9 (11)	4 (12)	5 (11)	0.87
Current tobacco smoker	34/77 (44)	15/32 (47)	19/45 (42)	0.69
Homeless	15 (19)	7 (21)	8 (17)	0.68
Ozone, 8-hr maximum^‡^ median (IQR), ppb	38 (34–43)	38 (34–43)	39 (35–45)	0.20
NO_2_, 1-hr maximum, median (IQR), ppb	49 (41–58)	52 (41–59)	49 (40–58)	0.70
PM_2.5_, 24-hr maximum, median (IQR), μg/m^3^	19.2 (15.7–23.0)	20.7 (16.4–24.0)	18.7 (14.9–22.7)	0.52

Abbreviations: PCP, *Pneumocystis* pneumonia; NO_2_, nitrogen dioxide; PM_2.5_, particulate matter < 2.5 μm in diameter

*Columns represent n (%) unless otherwise indicated

^†^Self-reported adherence to therapy at the time of enrollment

^‡^Maximum 8-hour concentration over the 14 days preceding hospitalization

### Ambient air pollutant exposures

Over the 14 days prior to hospitalization maximum 8-hr O_3_ levels ranged from 19–55 ppb (median 38, IQR 34–43); maximum 1-hr NO_2_ levels ranged from 20–93 ppb (median 49, IQR 41–58); and maximum 24-hr PM_2.5_ levels ranged from 6.1–49.8 μg/m^3^ (median 19.2, IQR 15.7–23.0) (Table 1). Air pollutant levels were similar for those with PCP compared to those without PCP.

### Serum and BALF antibody responses to Pneumocystis

The majority of patients exhibited detectable BALF IgA responses to Msg: 52% had detectable responses to MsgA, 90% to MsgC1, and 89% to MsgC8 (Table 2). However, only 8.6–26% of patients had detectable BALF IgM responses to Msg, and only 10–48% of patients had detectable BALF IgG responses to Msg. Serum and BALF antibody responses to Msg were positively correlated (r = 0.30–0.86 for IgM and 0.13–0.56 for IgG) with the strongest correlations seen for MsgA, MsgB, and MsgC1. Serum IgM and IgG antibody responses to Msg were highly predictive of BALF antibody responses to Msg in multivariable analyses, with the exception of IgG responses to MsgC8 and MsgC9.

**Table 2:** Serum and BALF antibody responses to Msg.

Antibody	Msg construct	Subjects with detectable antibody, n (%)		Mean (SD) antibody level, Units*		Correlation and linear regression analyses comparing serum to BALF antibody responses for each Msg construct^§§^		
		BALF	Serum	BALF	Serum	Correlation^†^ *r*	Effect estimate β^‡^^§^ (95%CI)	P value
		n = 81	n = 78**	n = 81	n = 78**			
IgA	MsgA	42 (52)	-	9.8 (25)	-	-	-	-
	MsgC1	73 (90)	-	16 (36)	-	-	-	-
	MsgC8	72 (89)	-	25 (39)	-	-	-	-
IgM	MsgA	18 (22)	74 (95)	8.5 (37)	60 (100)	0.86	**0.32 (0.28, 0.37)**	**<0.001**
	MsgB	13 (16)	75 (96)	2.7 (17)	32 (64)	0.67	**0.18 (0.13, 0.23)**	**<0.001**
	MsgC1	12 (15)	76 (97)	2.2 (11)	30 (28)	0.35	**0.14 (0.06, 0.23)**	**0.002**
	MsgC3	10 (12)	51 (65)	0.96 (4.1)	6.0 (8.9)	0.35	**0.18 (0.08, 0.29)**	**0.001**
	MsgC8	7 (8.6)	69 (88)	1.2 (6.1)	12 (15)	0.35	**0.14 (0.06, 0.23)**	**0.002**
	MsgC9	21 (26)	74 (95)	3.7 (16)	23 (23)	0.30	**0.23 (0.07, 0.39)**	**0.006**
IgG	MsgA	39 (48)	72 (92)	25 (54)	118 (112)	0.53	**0.30 (0.20, 0.40)**	**<0.001**
	MsgB	8 (10)	38 (49)	2.2 (8.5)	14 (24)	0.54	**0.19 (0.13, 0.26)**	**<0.001**
	MsgC1	14 (17)	70 (90)	11 (26)	54 (48)	0.56	**0.24 (0.13, 0.34)**	**<0.001**
	MsgC3	22 (27)	71 (91)	25 (57)	189 (203)	0.29	**0.08 (0.02, 0.15)**	**0.02**
	MsgC8	21 (26)	60 (77)	13 (30)	46 (67)	0.13	0.07 (-0.04, 0.19)	0.19
	MsgC9	24 (30)	59 (76)	29 (64)	85 (80)	0.21	0.14 (-0.03, 0.32)	0.11

Abbreviations: Msg, recombinant *Pneumocystis jirovecii* major surface glycoprotein; BALF, bronchoalveolar lavage fluid.

*BALF was diluted 1:5, and serum 1:100 prior to analysis. Values reported are uncorrected for the dilution factor

^†^Pearson correlation *r* between serum and BALF antibody response to each Msg construct

^‡^The change of BALF antibody responses to Msg (Units) predicted by a 1 Unit increase in serum antibody responses to Msg

^§^Multivariable linear regression adjusted for potential confounders—age, gender, race, CD4 count, viral load, antiretroviral therapy, PCP prophylaxis, prior PCP, current tobacco smoking, and homelessness—that were significant at a level of *P* < 0.2 in bivariate analyses.

**Missing serum specimens for 3 participants

^§§^Patients without detectable antibody responses were excluded from the analyses

### Immune characteristics and BALF IgA responses to Pneumocystis

We evaluated mean IgA responses to Msg constructs by CD4 count, viral load, antivirals, and PCP prophylaxis, finding that taking antiretrovirals and/or PCP prophylaxis was associated with statistically significantly higher mean IgA responses to Msg (S1 Table).

### Association of prior and current PCP with BALF IgA responses to Pneumocystis

Both a current diagnosis of PCP and a prior history of PCP were associated with BALF IgA responses to Msg. When stratifying by a current diagnosis of PCP, IgA responses to Msg were generally higher in those with a prior history of PCP compared to those with no prior history of PCP (Table 3). For instance, among patients without current PCP, those with a prior PCP history had a median IgA response to MsgC1 of 73.2 U (IQR 19.2–169) compared to 5.00 U (3.52–12.6) in those without prior PCP. Similar findings were evident for IgA responses to MsgA and MsgC8, but were statistically significant only for MsgC1 (*P* = 0.007).

**Table 3:** BALF IgA responses to Msg by current and prior PCP.

Msg construct	IgA Response, median (IQR), Units						
	Total	No current PCP			Current PCP		
		No prior PCP	Prior PCP	Total	No prior PCP	Prior PCP	Total
	n = 81	n = 30	n = 4	n = 34	n = 42	n = 5	n = 47
MsgA	1.05 (0–5.29)	1.12 (0–14.5)	15.7 (5.30–33.1)	1.40 (0–17.3)	0.53 (0–3.09)	0 (0–3.75)	0 (0–3.75)
MsgC1	5.32 (3.52–11.2)	**5.00 (3.52–12.6)**	**73.2 (19.2–169)**	5.26 (3.82–25.2)	5.32 (2.37–8.63)	6.69 (4.12–7.86)	5.34 (2.37–8.63)
MsgC8	13.3 (4.93–25.4)	11.9 (4.87–41.4)	22.5 (18.5–27.4)	13.8 (5.81–31.8)	12.0 (2.84–19.3)	31.5 (8.43–44.5)	12.8 (4.25–22.6)

Abbreviations: PCP, *Pneumocystis* pneumonia; Msg, recombinant *Pneumocystis jirovecii* major surface glycoprotein

Additionally, a current diagnosis of PCP was associated with decreased BALF IgA responses to Msg in linear regression analyses (Table 4). Again, these findings were most significant for IgA responses to MsgC1. For instance, in unadjusted analysis, a current diagnosis of PCP was associated with a 22.5 U lower BALF IgA response to MsgC1 compared to those individuals without current PCP; β -22.5, 95% CI -39.2, -5.82, *P* = 0.009). Findings were consistent in adjusted regression analyses, but with reduced statistical significance. Stratifying by prior PCP, the effect of current PCP on IgA responses to MsgC1 was greater (more negative) in those with a prior history of PCP compared to those without prior PCP (β -85.0 vs. -13.7, *P* interaction = 0.003). However, statistically significant effect modification was not seen for IgA responses to MsgA or MsgC8.

**Table 4:** Effect of current PCP on BALF IgA responses to Msg, stratified by a prior history of PCP.

Msg construct	Regression β coefficient* (95%CI), Units				
	All patients, unadjusted	All patients, adjusted^†^	No prior PCP	Prior PCP	*P* interaction
MsgA	-18.4 (-36.9, 0.21)	-7.88 (-26.6, 10.9)	-16.4 (-37.3, 4.44)	-25.2 (-53.4, 3.06)	0.53
MsgC1	**-22.5 (-39.2, -5.82)**	-17.3 (-37.3, 2.67)	**-13.7 (-27.3, -0.15)**	**-85.0 (-171, 1.16)**	**0.003**
MsgC8	-9.64 (-28.5, 9.24)	-2.45 (-22.9, 18.0)	-11.4 (-32.7, 9.95)	5.28 (-16.9, 27.5)	0.57

Abbreviations: PCP, *Pneumocystis* pneumonia; Msg, recombinant *Pneumocystis jirovecii* major surface glycoprotein

*The change in antibody response to Msg (Units) predicted by a current diagnosis of PCP

^†^Multivariable linear regression adjusted for potential confounders—age, gender, race, CD4 count, viral load, antiretroviral therapy, PCP prophylaxis, prior PCP, current tobacco smoker, and homeless—that were significant at *P* < 0.2 in bivariate analyses.

### Association of air pollutant exposures with BALF IgA responses to Pneumocystis

**Fig 2:**
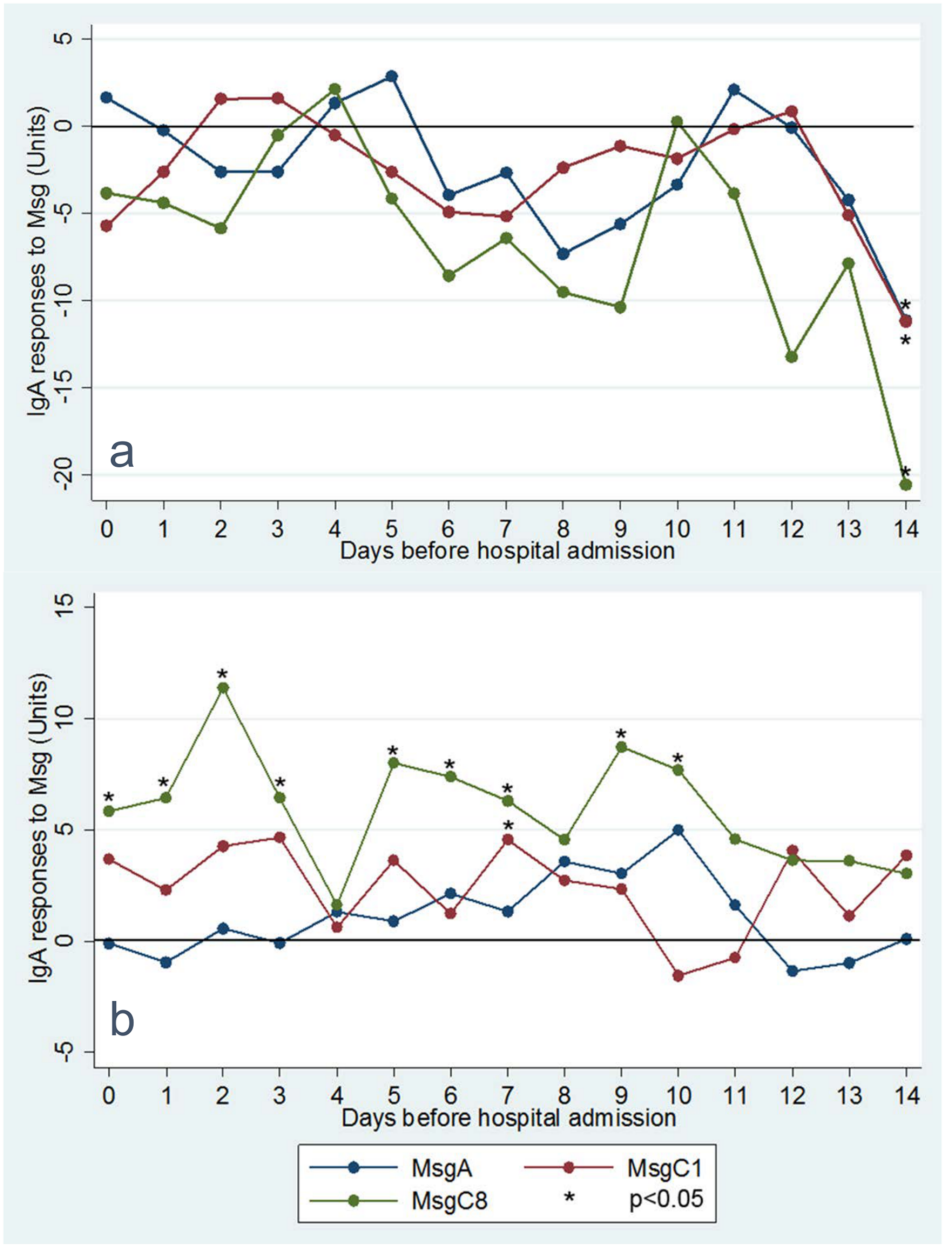
Bronchoalveolar IgA responses to MsgA, MsgC1, and MsgC8 predicted by a 10 ppb increase in (a) daily 8-hr maximum ozone and (b) daily 1-hr maximum nitrogen dioxide on each of the 14 days prior to hospital admission. Each point indicates a regression β coefficient estimating the IgA response to Msg for every 10 ppb increase in pollutant. Asterisked points represent statistically significant (*P* < 0.05) regression β coefficients. Multivariable Tobit regression analyses adjusted for potential confounders—age, gender, race, CD4 count, viral load, antiretroviral therapy, PCP prophylaxis, prior PCP, current tobacco smoker, and homelessness—that were significant at a level of *P* < 0.2 in bivariate analyses were used to calculate regression β coefficients. Abbreviations: PCP, *Pneumocystis* pneumonia; Msg, recombinant *Pneumocystis jirovecii* major surface glycoprotein.

Ambient air pollutant exposures in the 14 days prior to hospital admission were associated with BALF IgA responses to Msg. Increased daily 8-hr maximum O_3_ exposures were associated with suppressed IgA responses to Msg (Fig 2a and S1a Fig). Findings were most significant for exposures two weeks prior to admission. For instance, each 10 ppb increase in daily 8-hr maximum O_3_ was associated with an 11.1 U reduction in IgA responses to MsgA (β -11.1, 95% CI -22.0, -0.23, *P* = 0.046), an 11.2 U reduction in IgA responses to MsgC1 (β -11.2, 95% CI -20.0, -2.4, *P* = 0.01), and a 20.6 U reduction in IgA responses to MsgC8 (β -20.6, 95% CI -31.7, -9.50, *P* < 0.001) at 14 days prior to admission. Conversely, NO_2_ exposures were associated with increased IgA responses to Msg, most statistically significant for IgA responses to MsgC1 at one week prior to hospital admission and IgA responses to MsgC8 at 2 days prior to hospital admission (Fig 2b and S1b Fig). For instance, each 10 ppb increase in daily 1-hr maximum NO_2_ was associated with an 11.4 U increase in IgA response to MsgC8 two days prior to patients’ admission (β 11.4, 95% CI 5.17, 17.6, *P* < 0.001). Current tobacco smoking, however, was not associated with statistically significant increases or decreases in BALF IgA responses.

### Association of air pollutant exposures and IgA responses with clinical outcomes

Neither maximum levels of NO_2_, O_3_, and PM_2.5_ nor current tobacco smoking were statistically significantly associated with length of hospital admission or 2-month mortality. Similarly, BALF IgA responses to the Msg constructs were not statistically significantly associated with these clinical outcomes (see Supporting Information, S1 Data Set).

## Discussion

In this prospective cohort study we investigated clinical and environmental influences on bronchoalveolar antibody responses to *P*. *jirovecii* Msg constructs. We found that serum antibody responses to Msg were predictive of bronchoalveolar responses, and that although most patients had BALF IgA responses to Msg, the majority did not have detectable BALF IgM or IgG responses. PCP was significantly associated with BALF IgA responses to Msg such that those with a prior history of PCP (and not current PCP) had elevated IgA responses and those with a current diagnosis of PCP had reduced IgA responses. Pre-admission ambient levels of O_3_ were associated with decreased IgA responses while NO_2_ was associated with increased BALF IgA responses to Msg.

There was a moderate correlation between BAL and serum antibody responses and a highly statistically significant association between BAL and serum antibody responses using multivariable linear regression analysis. The correlation and level of significance could be strong enough to be clinically relevant. Our study is hypothesis generating, and more studies are needed to evaluate the clinical utility of antibody responses to *Pneumocystis* not only in serum but also in BALF.

Those with a prior history of PCP had higher IgA responses to *Pneumocystis* Msg. This suggests immunologic memory to prior *Pneumocystis* infection. Furthermore, IgA responses were present even in those without prior or current PCP, suggesting immunologic memory to prior subclinical *P*. *jirovecii* infection or colonization, or perhaps IgA responses to current subclinical infection or colonization. Prior studies have found that up to 85% of humans are colonized with *P*. *jirovecii* during early childhood with many converting during mild respiratory illnesses thought to be *Pneumocystis* infections [37, 38]. In another study of hospitalized HIV-infected patients with non-*Pneumocystis* pneumonia, 68% were colonized with P. *jirovecii* [39].

Those with current PCP experienced lower BALF antibody responses to Msg compared to those without a current diagnosis of PCP, similar to previously reported findings [30]. The mechanisms for lower IgA responses to Msg in HIV-infected patients with PCP are unclear. IgA is a first-line humoral defense against airway pathogens and it is conceivable that low IgA responses were a predisposing factor for PCP development. Secretory IgA binds to airspace pathogens to facilitate clearance. Thus, it is also possible that binding of IgA to the high burden of *P*. *jirovecii* in the airways and alveoli of patients with current PCP outstripped IgA production. Detectable IgA responses to Msg were present in the majority of patients whereas IgM and IgG responses to Msg were less common. This likely reflects the larger size of IgM and IgG with the resultant difficult passage into airways except by means of transudation of blood fluids into inflamed airways.

Immune status plays an important role in antibody responses to *Pneumocystis*. In a large prospective cohort study of hospitalized patients with suspected pulmonary infection in Kampala, Uganda, we found statistically significantly lower serum IgM responses to MsgC1, MsgC3, MsgC8, and MsgC9 in patients infected with HIV compared to those not infected, and among those with HIV, CD4 <200 also predicted lower IgM responses [25]. In the current study in San Francisco, we found that, in unadjusted analyses, antiretroviral use was associated with increased IgA responses to Msg, possibly through an immune reconstitution, though we did not find CD4 or viral load to be statistically significantly associated with IgA responses. Similarly, PCP prophylaxis was associated with higher IgA responses to Msg, perhaps through prevention of PCP organisms in the airway that would lead to consumption of secretory IgA.

This is the first published study to our knowledge to examine the impact of air pollutants on bronchoalveolar antibody responses to *Pneumocystis*. Ambient O_3_ levels in the 2 weeks prior to admission for respiratory illness were associated with a trend towards decreased BALF IgA responses to Msg, while NO_2_ levels were associated with a trend towards increased responses. It is unclear why the two pollutants were associated with antibody responses in opposite directions. One possible explanation is that air pollutants can have both inflammatory and suppressive effects on the immune system as evidenced in numerous studies. O_3_ is a potent oxidant resulting in bronchoalveolar [40, 41] and systemic [42, 43] inflammation. Nevertheless, consistent with our findings, animal studies have found that rats in the first 2 weeks of exposure to O_3_ experienced decreased antibody responses to microbial antigen such as *Listeria* antigen [44]. NO_2_ is also pro-inflammatory but animal and human exposure studies have found mixed effects of NO_2_ inhalation on bronchoalveolar and systemic antibody responses [45–49]. A recent study of ours found increased ambient levels of NO_2_ to be associated with suppressed serum IgM responses to *Pneumocystis* Msg, whereas IgG responses were not suppressed [28]. Tobacco smoke, which contains NO_2_ along with numerous other toxic compounds [50], has been found to be associated with decreased serum IgG responses to *Pneumocystis* Msg in a prior study [51]. Our study is the first to evaluate the association of smoking with BALF antibody responses to Msg, and no statistically significant associations were evident. However, cotinine levels were not available to verify and quantify tobacco smoke exposure. Although the trends of air pollutant associations with BALF IgA levels observed in this small prospective cohort study are intriguing, with the small sample size and high *P* values the findings remain inconclusive and further studies are needed in this area. San Francisco air pollutant levels are relatively low compared to other large urban areas [52] and it is possible that more pronounced effects would be seen in more heavily polluted areas.

Finally, neither air pollutant levels prior to admission nor BALF IgA responses to *Pneumocystis* Msg were associated with adverse clinical outcomes as measured by prolonged length of hospital admission and mortality. However, our small sample size with few deaths make it difficult to interpret these findings.

## Conclusions

Prior studies have shown that humoral immunity plays an important role in host defense against *P*. *jirovecii*. In our study we found that both clinical and environmental factors were associated with BALF IgA responses to *Pneumocystis* Msg. IgA was increased in those with a prior history of PCP yet decreased in those with current PCP, findings suggestive of immunologic memory and possible airway consumption during active infection, though small numbers in these subgroups limit the generalizability of these findings. Ozone levels within the two weeks prior to hospital admission were associated with decreasing trends of IgA responses to Msg, while NO_2_ levels were associated with increasing trends of IgA to Msg. Although air pollutant and BALF IgA responses to Msg were not associated with clinical outcomes in this prospective cohort, the sample size was small and air pollutant levels low, and further studies are needed to evaluate the clinical significance of environmental influences on lung immunity against *P*. *jirovecii*.

## Supporting information

S1 Fig Bronchoalveolar IgA responses to (1) MsgA, (2) MsgC1, and (3) MsgC8 for every 10 ppb/m^3^ increase in (a) daily 8hr-maximum ozone and (b) daily 1hr maximum nitrogen dioxide. Points represent regression β coefficient point estimates and bars represent 95% CIs. Multivariable Tobit regression analyses adjusted for potential confounders—age, gender, race, CD4 count, viral load, antiretroviral therapy, PCP prophylaxis, prior PCP, current tobacco smoker, and homelessness—that were significant at a level of *P* < 0.2 in bivariate analyses were used to calculate regression β coefficients and 95% CIs.
(PDF)

S1 Table Unadjusted mean IgA responses to Msg by immune status and treatment. (DOCX)

S1 Data Set Minimal data set. (XLSX)
