# Supplementary material for: Effects of clinical and environmental factors on bronchoalveolar antibody responses to Pneumocystis jirovecii: A prospective cohort study of HIV+ patients
Source: PLoS One. 2017 Jul 10;12(7):e0180212. doi: 10.1371/journal.pone.0180212 (PMC5503245; doi:10.1371/journal.pone.0180212)
Supplement: S1 Fig — Points represent regression β coefficient point estimates and bars represent 95% CIs. Multivariable Tobit regression analyses adjusted for potential confounders—age, gender, race, CD4 count, viral load, antiretroviral therapy, PCP prophylaxis, prior PCP, current tobacco smoker, and homelessness—that were significant at a level of P < 0.2 in bivariate analyses were used to calculate regression β coefficients and 95% CIs. (PDF) [file pone.0180212.s001.pdf]

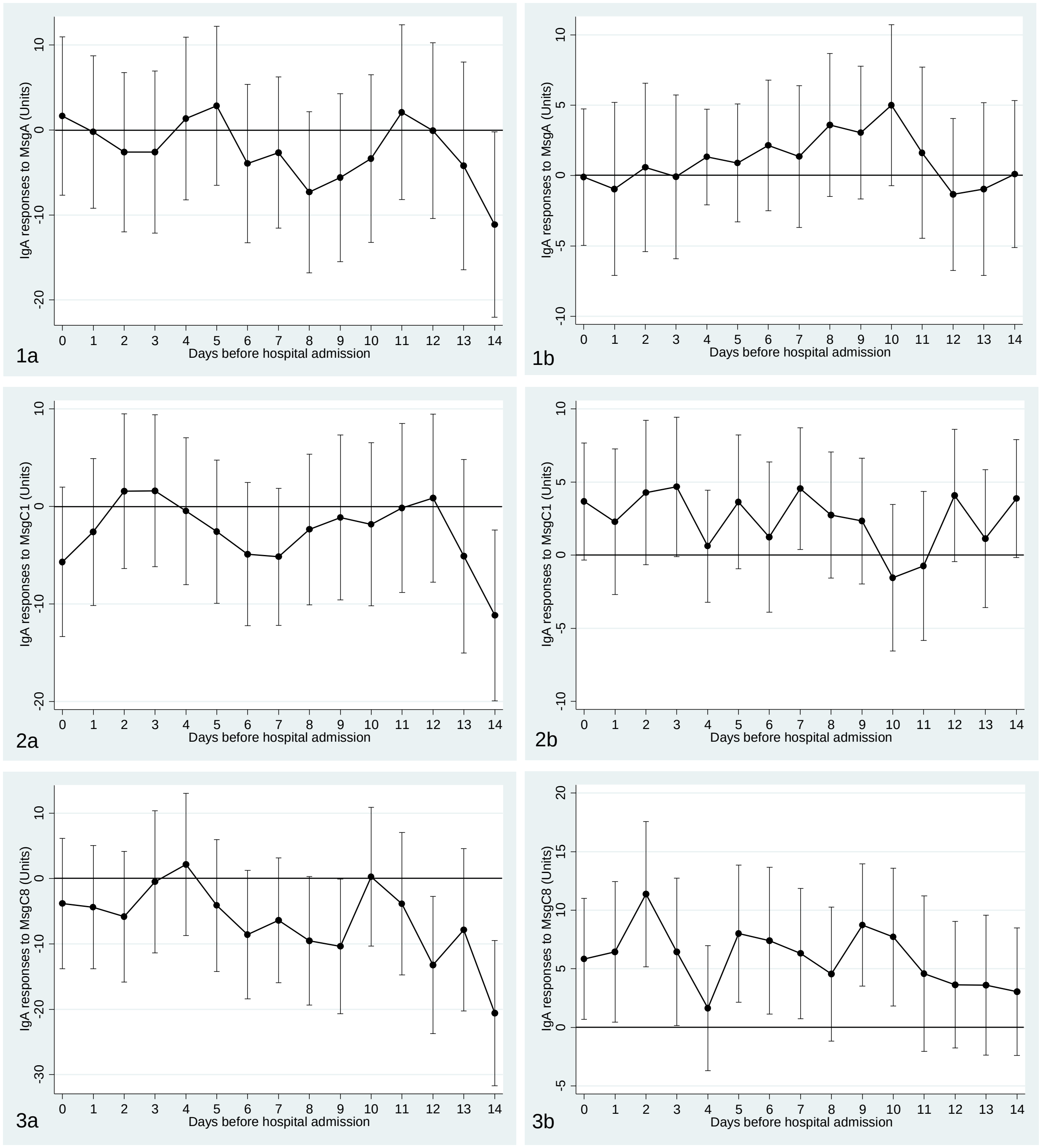

S1 Fig. Bronchoalveolar IgA responses to (1) MsgA, (2) MsgC1, and (3) MsgC8 for every 10 ppb/m^3 increase in (a) daily 8hr-maximum ozone and (b) daily 1hr-maximum nitrogen dioxide. Points represent regression β coefficient point estimates and bars represent 95% CIs. Multivariable Tobit regression analyses adjusted for potential confounders—age, gender, race, CD4 count, viral load, antiretroviral therapy, PCP prophylaxis, prior PCP, current tobacco smoker, and homelessness—that were significant at a level of $P < 0.2$ in bivariate analyses were used to calculate regression β coefficients and 95% CIs.
