# Supplementary material for: Effects of clinical and environmental factors on bronchoalveolar antibody responses to Pneumocystis jirovecii: A prospective cohort study of HIV+ patients
Source: PLoS One. 2017 Jul 10;12(7):e0180212. doi: 10.1371/journal.pone.0180212 (PMC5503245; doi:10.1371/journal.pone.0180212)
Supplement: S1 Table — (DOCX) [file pone.0180212.s002.docx]

Supplemental Material

**S1 Table. Unadjusted mean IgA responses to Msg by immune status and treatment**

| Immune characteristic | | N | Mean IgA response to Msg construct, U (SD) | | |
| --- | --- | --- | --- | --- | --- |
|  |  |  | MsgA | MsgC1 | MsgC8 |
| CD4 > 200 | No | 63 | 11.8 (30.0) | 19.4 (40.6) | 27.7 (42.5) |
|  | Yes | 18 | 4.70 (11.0) | 6.71 (4.2) | 15.0 (38.8) |
|  | *P* value | -- | 0.30 | 0.19 | 0.22 |
| Viral load > 126,000 copies/ml (median) | No | 40 | 13.9 (33.3) | 14.9 (25.7) | 24.8 (44.8) |
|  | Yes | 41 | 6.68 (25.3) | 18.3 (44.4) | 24.9 (32.3) |
|  | *P* value | -- | 0.20 | 0.68 | 0.99 |
| Taking antiretrovirals | No | 48 | 6.55 (12.7) | **9.27 (13.4)** | 20.1 (31.8) |
|  | Yes | 28 | 13.9 (35.5) | **28.6 (55.8)** | 28.1 (38.0) |
|  | *P* value | -- | 0.20 | **0.02** | 0.33 |
| Taking PCP prophylaxis | No | 66 | **6.90 (14.9)** | **13.1 (30.6)** | **19.8 (30.2)** |
|  | Yes | 13 | **27.0 (51.8)** | **36.4 (56.0)** | **52.6 (63.6)** |
|  | *P* value | -- | **0.008** | **0.04** | **0.005** |
